# C5 Palsy After Cervical Decompression: A 10-Year Single-Institution Study Highlighting Early Onset, High Bilaterality, and Poor Recovery

**DOI:** 10.7759/cureus.102781

**Published:** 2026-02-01

**Authors:** Sargunan B, Vishnu Prasath, Karthik Sudhakar, Thomas John

**Affiliations:** 1 Spine Surgery, SKS Hospital and Postgraduate Medical Institute, Salem, IND; 2 Orthopedic Surgery, Periyar Government Hospital, Chennai, IND

**Keywords:** c5 nerve root palsy, cervical decompression surgery, cervical spine, postoperative complication, spine surgery

## Abstract

Introduction: C5 palsy is a recognized complication following cervical decompression procedures, presenting as new-onset deltoid or biceps weakness. Despite extensive investigation, its mechanisms remain debated, involving root traction, foraminal stenosis, and possible cord-level ischemia-reperfusion injury. Reported incidences vary widely, and recovery outcomes differ across populations, making institutional data valuable.

Study: This retrospective observational study evaluated postoperative C5 palsy over a 10-year period in patients undergoing cervical decompression. All cases were assessed for incidence, timing of onset, laterality, surgical approach, and long-term neurological outcomes. Additional emphasis was placed on identifying patterns that differ from those in the existing literature, particularly regarding severity and bilaterality.

Results: Among 198 cervical decompressions, 14 patients (7.1%) developed C5 palsy. Early onset was universal, with 57.1% (eight patients) occurring immediately postoperatively. Bilateral involvement was unusually high at 35.7% (five patients). Eight patients (57.1%) exhibited persistent deficits, while three patients (21.4%) worsened; only three patients (21.4%) showed improvement, and none recovered full strength. Posterior approaches accounted for 71.4% of cases, and foraminal stenosis was present in five patients (35.7%).

Conclusion: C5 palsy in this cohort showed early onset, high bilaterality, and poor recovery, suggesting multifactorial mechanisms rather than isolated root traction. Posterior approaches and foraminal stenosis appear to be important risk factors. Preventive strategies should include detailed imaging, selective foraminotomy, neuromonitoring, and refined surgical technique. Further multicenter studies are needed to clarify pathogenesis and improve outcomes.

## Introduction

Cervical decompression procedures, including anterior cervical discectomy and fusion, laminoplasty, and laminectomy with or without fusion, are widely performed for degenerative cervical myelopathy and ossification of the posterior longitudinal ligament. Although these surgeries are effective in halting neurological decline, they may be complicated by postoperative neurological deficits, among which C5 palsy remains one of the most clinically significant. C5 palsy is classically defined as new-onset deltoid and/or biceps weakness without lower-limb deterioration [1]. Reported incidence varies considerably across studies, ranging from 1% to more than 20%, influenced by surgical approach, baseline pathology, and definition used. Recent meta-analyses estimate a pooled incidence of 5-8%, highlighting its continued relevance in cervical spine surgery [2]. Despite extensive investigation, the pathogenesis of C5 palsy remains incompletely understood. Proposed mechanisms include traction injury to the C5 nerve root due to posterior cord drift following decompression, pre-existing C4-C5 foraminal stenosis becoming functionally significant postoperatively, and ischemia-reperfusion injury targeting anterior horn cells at the C5 segment [3]. Although many patients experience partial or complete recovery, a substantial minority develop persistent or severe deficits, leading to long-term disability [4]. Owing to this variability in presentation and outcome, long-term institutional data are essential to better characterize incidence patterns, identify high-risk subgroups, and guide preventive strategies. The present study contributes to this knowledge gap by analyzing a 10-year series of cervical decompressions to evaluate the incidence, onset, laterality, and long-term outcomes of postoperative C5 palsy, with comparison to established literature.

## Materials and methods

This retrospective observational study was conducted at SKS Hospital, Salem, India, and included all patients who underwent cervical decompression surgery between June 2015 and June 2025. The aim of this study was to evaluate the occurrence and clinical characteristics of postoperative C5 palsy in patients undergoing cervical decompression surgery at a single tertiary care institution over a 10-year period. The primary objective was to determine the incidence of C5 palsy and to describe its temporal onset, laterality, and neurological recovery pattern using standardized clinical definitions and longitudinal follow-up. The secondary objectives were to characterize the distribution of C5 palsy across different surgical approaches and pathologies, to document associated radiological features such as C4-C5 foraminal stenosis, and to compare observed descriptive trends with existing literature to generate hypotheses regarding potential mechanisms and preventive considerations, without inferring causality. Patients were identified from operative registries, electronic medical records, and departmental surgical logs. All forms of cervical decompression were included: anterior cervical discectomy and fusion (ACDF), corpectomy, posterior laminectomy with or without instrumented fusion, and laminoplasty. The inclusion criteria encompassed adult patients (≥18 years) undergoing decompression for cervical spondylotic myelopathy (CSM), ossification of the posterior longitudinal ligament (OPLL), multilevel canal stenosis, or other degenerative pathologies. Patients were excluded if they had preoperative deltoid or biceps weakness, brachial plexus lesions, traumatic cervical root avulsion, prior cervical surgery at the index level, acute spinal cord injury requiring emergent decompression, intraoperative neurological complications unrelated to C5 palsy, or incomplete postoperative follow-up. C5 palsy was defined as new-onset postoperative weakness of the deltoid and/or biceps by at least one Medical Research Council (MRC) grade compared to the preoperative baseline, in the absence of new lower limb motor deterioration, new sensory loss unrelated to C5 dermatome, or evidence of systemic conditions such as electrolyte abnormalities that could confound neurological assessment [2]. This definition was chosen to maintain comparability with established literature and previously validated diagnostic criteria used in major clinical series [2,5].

Preoperative evaluation included detailed neurological assessment, MRC grading of all limb muscles, and radiological imaging comprising MRI of the cervical spine for canal stenosis, signal changes, and cord morphology, along with CT scans when required to evaluate foraminal stenosis or OPLL burden. Surgical approach selection was made based on pathology, number of vertebral levels involved, sagittal alignment, and surgeon preference. For posterior surgery, a laminectomy was performed with preservation of facet joints whenever possible, and lateral mass screws were placed using standard entry points and trajectories. Laminoplasty was carried out using an open-door technique when indicated. Prophylactic foraminotomy at C4-C5 was not routinely performed, nor was intraoperative neuromonitoring (IONM) consistently available during the 10-year study period. Intraoperative parameters, including the number of decompressed levels, duration of surgery, instrumentation use, blood loss, anesthetic records, intraoperative complications, and technical variations, were recorded. Postoperative neurological examinations were conducted immediately in the recovery room, at six and 24 hours, daily until discharge, and at each outpatient follow-up visit (one month, three months, six months, 12 months, and annually thereafter). Timing of C5 palsy onset was classified as immediate (within 24 hours), early (24-48 hours), delayed (3-7 days), or late (8-14 days). Laterality was classified as unilateral (right or left) or bilateral. Postoperative MRI and electromyography (EMG)/nerve conduction study (NCS) were obtained selectively based on clinical progression, worsening symptoms, or lack of early recovery. MRI scans were used primarily to rule out compressive causes such as postoperative hematoma, residual stenosis, instrumentation malposition, or cord edema. EMG/NCS performed at three to six weeks helped differentiate radiculopathy from anterior horn cell or cord-level injury. For risk factor analysis, all procedures involving a posterior component, including posterior-only and combined anterior-posterior approaches, were grouped together as posterior involvement.

Management strategies for C5 palsy were primarily conservative and included physiotherapy focused on shoulder abduction, elbow flexion, scapular stabilization, and deltoid strengthening. Oral analgesics, muscle relaxants, and supportive measures were provided as needed. Systemic corticosteroids were not routinely administered, reflecting the absence of strong evidence for benefit [5]. Surgical re-exploration or delayed foraminotomy was reserved for patients with progressive weakness accompanied by radiologically confirmed foraminal stenosis. Recovery status was documented using MRC grading at each visit. Final outcomes were categorized as improved (≥1 MRC grade increase), persistent (no change), or worsened (≥1 MRC grade decline), with follow-up extending to a minimum of one year for all included patients. Statistical analysis was performed using descriptive statistics for demographic and clinical variables. Continuous variables were summarized as mean ± standard deviation, while categorical variables were represented as percentages. Chi-square test was used to compare categorical variables. SPSS version 31.0.1.0 software (IBM Corp., Armonk, NY) was used for statistical analysis. A p-value of <0.05 was considered statistically significant.

## Results

During the 10-year study period, a total of 198 cervical decompression surgeries were performed. Among these, 14 patients developed postoperative C5 palsy, corresponding to an incidence of 7.1%. All 14 affected patients were male (100%), with a mean age of 62.4 years (range = 47-75 years). This demographic distribution indicates an older male-predominant cohort aligned with known risk factors. Furthermore, 11 of the 14 patients (78.6%) had CSM or OPLL, while three patients (21.4%) presented with other degenerative cervical pathologies (Table 1).

**Table 1:** Incidence and patient demographics. C5: cervical root 5; CSM: cervical spondylotic myelopathy; OPLL: ossification of the posterior longitudinal ligament; N: number of patients; %: percentage of patients who had C5 palsy.

Parameter	Value, N (%)
Total surgeries	198
C5 palsy cases	14
Mean age	62.4 years
Age range	47–75 years
Male	14 (100%)
CSM/OPLL	11 (78.6%)
Other diagnoses	3 (21.4%)

Posterior decompression was the most frequently performed surgical approach in patients who developed C5 palsy. A total of 10 out of 14 patients (71.4%) underwent posterior laminectomy or laminoplasty. Combined anterior-posterior procedures accounted for three cases (21.4%), while an isolated anterior approach was used in one case (7.1%). The median number of decompressed levels was three (range = 2-5). Instrumented fusion was carried out in 10 patients, reflecting a high rate of stabilization procedures in the cohort (Table 2). The C4-C5 segment was the most frequently decompressed level among patients who developed C5 palsy, either as a single-level procedure or as part of multilevel cervical decompression constructs. Other potential risk factors, including age, underlying diagnosis (CSM versus OPLL), number of decompressed levels, and laterality of surgery, were examined; however, no consistent or statistically meaningful patterns were identified within this cohort. Although a range of cervical levels was operated upon, involvement of the C4-C5 level was a consistent denominator in the majority of affected cases.

**Table 2:** Surgical approach characteristics (total number of C5 palsy cases = 14).

Parameter	Value, N (%)
Posterior approach	10 (71.4%)
Anterior approach	1 (7.1%)
Combined approach	3 (21.4%)
Median levels decompressed	3 (2–5)
Instrumented fusion	10 cases

All cases of postoperative C5 palsy occurred within 14 days of surgery. A majority of patients (8, 57.1%) developed weakness immediately in the postoperative recovery period (<24 hours). Three patients (21.4%) had onset within 24-48 hours, while two patients (14.3%) developed symptoms between days three and seven. Only one patient (7.1%) experienced onset between days eight and 14 (Table 3). This demonstrates an overwhelmingly early onset pattern, with nearly 80% of cases presenting within the first 48 hours.

**Table 3:** Timing of onset of C5 palsy (total number of C5 palsy cases = 14). N: number of patients.

Parameter	Value, N (%)
Immediate (<24 hours)	8 (57.1%)
24–48 hours	3 (21.4%)
3–7 days	2 (14.3%)
8–14 days	1 (7.1%)

Laterality analysis showed that five of 14 patients (35.7%) developed bilateral C5 palsy, a proportion higher than that reported in large multicenter studies, where bilateral involvement is generally described as uncommon. Among unilateral cases, four patients (28.6%) had right-sided involvement and five patients (35.7%) had left-sided involvement. Bilateral involvement was associated with more severe functional deficits and poorer recovery outcomes based on follow-up MRC grading (Table 4).

**Table 4:** Laterality of C5 palsy (total number of C5 palsy cases = 14).

Parameter	Value
Bilateral palsy	5 (35.7%)
Right-sided palsy	4 (28.6%)
Left-sided palsy	5 (35.7%)

At a mean follow-up duration of 2.5 years (range = 1-4 years), only three out of 14 patients (21.4%) demonstrated improvement of at least one MRC grade. A majority of patients (8, 57.1%) showed persistent deficits with no meaningful improvement, while three patients (21.4%) experienced worsening weakness when compared with their initial postoperative assessment. Notably, none of the patients, including those with unilateral palsy, regained full preoperative muscle strength. Bilateral cases demonstrated particularly poor recovery, consistent with more severe neurological involvement (Table 5).

**Table 5:** Recovery outcomes (total number of C5 palsy cases = 14). MRC: Medical Research Council scale for muscle strength; N: number of patients.

Parameter	Value, N (%)
Improved (≥1 MRC)	3 (21.4%)
Persistent deficit	8 (57.1%)
Worsened	3 (21.4%)
Full recovery	0 (0%)

Among unilateral cases (n = 9), improvement occurred in two patients (22.2%), persistence in five patients (55.6%), and worsening in two patients (22.2%). Among bilateral cases (n = 5), improvement was seen in one patient (20.0%), persistence in three patients (60.0%), and worsening in one patient (20.0%). Total outcome distribution showed 21.4% improvement, 57.1% persistence, and 21.4% worsening (Table 6). Descriptive analysis using chi-square testing did not demonstrate a statistically significant association between laterality and outcome, recognizing the limitations imposed by the small sample size.

**Table 6:** Laterality vs. outcomes. n: number of patients; X^2^:chi-square value.

Laterality	Improved	Persistent	Worsened	Chi-square value	P-value
Unilateral (n=9)	2 (22.2%)	5 (55.6%)	2 (22.2%)		
Bilateral (n=5)	1 (20.0%)	3 (60.0%)	1 (20.0%)		
Total (n=14)	3 (21.4%)	8 (57.1%)	3 (21.4%)	X^2^=0.0259	0.9871

For posterior approach cases (n = 10), improvement occurred in two patients (20.0%), persistence in six patients (60.0%), and worsening in two patients (20.0%). For non-posterior approaches (n = 4), improvement occurred in one patient (25.0%), persistence in two patients (50.0%), and worsening in one patient (25.0%). Total outcomes showed improvement in three patients (21.4%), eight patients (957.1%) had persistent deficit, and three patients (21.4%) worsened. Chi-square analysis demonstrated no significant association between surgical approach and outcome (Table 7).

**Table 7:** Surgical approach vs. outcomes. n: number of patients; X^2^: chi-square value.

Surgical approach	Improved	Persistent	Worsened	Chi-square value	P-value
Posterior (n=10)	2 (20.0%)	6 (60.0%)	2 (20.0%)		
Non-posterior (n=4)	1 (25.0%)	2 (50.0%)	1 (25.0%)		
Total (n=14)	3 (21.4%)	8 (57.1%)	3 (21.4%)	X^2^=0.1167	0.9433

## Discussion

Postoperative C5 palsy remains one of the most challenging complications of cervical decompression surgery, with an etiology that continues to be debated. In the present study, the incidence of C5 palsy was 7.1% (14 out of 198 patients), aligning with reported ranges of approximately 5-8% in large systematic reviews [6]. However, several features of this cohort differ from commonly published series. Most notably, bilateral palsy occurred in five patients (35.7%), which is significantly higher than the generally rare bilateral involvement described in multicenter studies [7]. The timing of onset also followed a consistent pattern seen in the literature, with eight patients (57.1%) developing immediate postoperative weakness, and all cases presenting within 14 days. This early onset supports the traction theory, wherein posterior shift of the spinal cord after decompression places tension on the short and horizontally oriented C5 root, a mechanism emphasized by Hitchon et al. [8]. Imagama et al. similarly demonstrated that posterior migration following multilevel laminectomy increases mechanical stress on the C5 root because it has the shortest root distance to the intervertebral foramen [9]. Yet, traction alone does not explain the unusually severe and bilateral deficits observed in our series, nor the poor recovery profile, in which only three patients (21.4%) showed any improvement.

Several authors have proposed that cord-level injury contributes substantially to C5 palsy and may underlie more severe forms. Bak et al. reported segmental T2 hyperintensity at the C5 region following laminoplasty and suggested that these findings may reflect ischemia-reperfusion injury to the vulnerable anterior horn cells rather than isolated radiculopathy [10]. Hofler et al. expanded this theory, demonstrating that patients with preoperative cord signal changes had significantly higher odds of severe postoperative deficits, implying intrinsic neuronal susceptibility [11]. This mechanism may be relevant to our population, where the predominance of older patients and the high prevalence of OPLL likely represent chronic ischemic stress, reducing the spinal cord’s capacity to tolerate abrupt decompression. In our cohort, five patients had documented preoperative C4-C5 foraminal stenosis, a well-known risk factor described by Yoshihara et al., who showed that narrowed foramina predispose the C5 root to compression once the spinal cord shifts posteriorly [12]. Thammongkolchai et al. demonstrated that prophylactic foraminotomy significantly reduces the incidence of C5 palsy in such patients [13], whereas Takase et al. reported increased axial pain and potential facet compromise with routine foraminotomy, recommending selective use instead [14]. Our institution did not routinely employ foraminotomy or standardized neuromonitoring during the study period, which may partly explain the higher severity of deficits observed.

The predominance of posterior involvement among C5 palsy cases (10 patients, 71.4%) is consistent with observations reported in previously published series [15]. Posterior decompression is associated with greater posterior cord drift, increased foraminal traction forces, and wider laminectomy windows at the C5 level, all of which contribute to C5 vulnerability. Oshina et al. reported a higher incidence of C5 palsy following posterior approaches compared with anterior procedures in pooled analyses [15]. Even though our statistical analysis did not yield a significant association between surgical approach and outcome, largely due to small subgroup sizes, the clinical trend remains informative. Recovery outcomes in the present study were notably poorer than in most published cohorts. While many studies report recovery within six to 12 months in the majority of patients, especially in unilateral cases, our findings showed only three patients (21.4%) improving, eight patients (57.1%) remaining unchanged, and three patients (21.4%) worsening, with none regaining full strength. Gu et al. reported that even persistent palsy may show slow recovery over several years [16], yet bilateral cases in our series exhibited particularly limited progression, consistent with more profound underlying neural injury.

These findings underscore the multifactorial and heterogeneous nature of C5 palsy. Root traction, foraminal stenosis, and reperfusion-related anterior horn susceptibility may all contribute in varying degrees depending on patient anatomy, preoperative spinal cord reserve, and surgical technique. Based on these observations and prior evidence, several preventive strategies should be considered. Detailed preoperative imaging, including CT to evaluate foraminal stenosis and MRI to assess cord signal changes, is crucial for identifying patients at higher risk. Selective prophylactic foraminotomy may be employed in those with significant stenosis, particularly when posterior multilevel decompression is planned. Although intraoperative neuromonitoring for C5 pathways remains imperfect in sensitivity, it may provide early warnings of root or cord perturbation. Finally, meticulous surgical technique, avoidance of excessive decompression at the C5 level, preservation of facet stability, and maintenance of adequate spinal cord perfusion may mitigate postoperative deficits. Definitive preventive strategies in our study could not be established due to the small sample size, heterogeneity of surgical approaches, and variability in underlying pathology. These findings suggest that individualized surgical planning and heightened postoperative neurological surveillance may currently be more practical than a single uniform preventive strategy.

Strengths of the study

The strengths of this study include its long-term 10-year single-institution dataset, which provides real-world insight into the incidence and clinical course of postoperative C5 palsy in routine practice. All cases were identified using a consistent and clearly defined diagnostic criterion, with standardized neurological assessment and longitudinal follow-up, enhancing internal descriptive validity. Detailed tabulation of timing of onset, laterality, surgical approach, and recovery patterns allows transparent interpretation of outcomes and comparison with existing literature. The uniform institutional setting, inclusion of consecutive cases, and representation of common pathologies such as cervical spondylotic myelopathy and OPLL further strengthen the reliability of the observed descriptive trends and support the study’s value as hypothesis-generating observational evidence.

Limitations of the study

This study has several important limitations that should be acknowledged. First, this is a retrospective, single-institution case series restricted to patients who developed postoperative C5 palsy, without comparison to the larger cohort of patients who did not develop this complication. As such, the study is not designed to identify independent risk factors, establish causality, or evaluate the effectiveness of preventive strategies, and its findings should be interpreted as descriptive rather than inferential. The observed incidence, early onset pattern, predominance of posterior approaches, high bilaterality, and poor recovery are supported by robust internal descriptive data but are based on small absolute numbers, limiting generalizability and precluding multivariate adjustment or causal modeling. Second, intraoperative techniques, including laminectomy, laminoplasty, and management of OPLL, were not standardized across the study period, and the use of intraoperative neuromonitoring and prophylactic foraminotomy was inconsistent and not guided by predefined criteria, which limits reproducibility and detailed technique-outcome correlation. Neurological assessment relied on routine clinical MRC grading and imaging interpretation documented in medical records, without formalized scoring tools, blinded assessment, or centralized imaging review, introducing potential observer variability. Although all patients had a minimum follow-up of one year, follow-up completeness beyond this threshold and ultra-long-term recovery could not be uniformly assessed. From a statistical perspective, the small sample size constrained analytical power; no sample size calculation was feasible, confidence intervals and effect sizes were not calculated, and subgroup analyses were underpowered, appropriately yielding non-significant associations despite observed trends. Consequently, conclusions regarding mechanisms such as multifactorial injury, the role of foraminal stenosis, or the influence of posterior approaches should be viewed as hypothesis-generating and aligned with existing literature rather than definitive evidence. Preventive recommendations are extrapolated from published data rather than direct interventional findings from this cohort. These limitations are inherent to retrospective observational designs and underscore the need for larger, multicenter prospective studies with standardized surgical protocols, imaging assessment, and long-term follow-up to more precisely define mechanisms and optimize prevention strategies.

Despite its limitations, the institutional incidence identified in this study provides a practical benchmark for postoperative counselling, internal audit, and early clinical vigilance. Awareness of an approximate 7% incidence of C5 palsy allows surgeons to better inform patients preoperatively, anticipate early neurological deficits, and initiate timely rehabilitation. Within the institution, these data may guide selective attention to high-risk scenarios such as posterior decompression involving the C4-C5 level and reinforce the importance of structured postoperative neurological surveillance.

## Conclusions

Postoperative C5 palsy remains a significant complication of cervical decompression, with this study showing a 7.1% incidence and an unusually high rate of bilateral involvement. With early onset in all cases and poor recovery outcomes, only three patients improved, and none regained full strength, suggesting that both root traction and cord-level mechanisms, such as ischemia-reperfusion, may contribute. The strong association with posterior approaches and preoperative foraminal stenosis highlights key risk factors. Preventive strategies, including selective foraminotomy, careful surgical technique, and standardized neuromonitoring, may reduce risk. Larger prospective studies are required to better define mechanisms and prevention.
